# *WRN*-Mutated Colorectal Cancer Is Characterized by a Distinct Genetic Phenotype

**DOI:** 10.3390/cancers12051319

**Published:** 2020-05-22

**Authors:** Kai Zimmer, Alberto Puccini, Joanne Xiu, Yasmine Baca, Gilbert Spizzo, Heinz-Josef Lenz, Francesca Battaglin, Richard M. Goldberg, Axel Grothey, Anthony F. Shields, Mohamed E. Salem, John L. Marshall, W. Michael Korn, Dominik Wolf, Florian Kocher, Andreas Seeber

**Affiliations:** 1 Department of Hematology and Oncology, Comprehensive Cancer Center Innsbruck, Innsbruck Medical University, 6020 Innsbruck, Austria; kai-christian.zimmer@student.i-med.ac.at (K.Z.); gilbert.spizzo@i-med.ac.at (G.S.); dominik.wolf@i-med.ac.at (D.W.); florian.kocher@i-med.ac.at (F.K.); 2 Norris Comprehensive Cancer Center, Keck School of Medicine, University of Southern California, Los Angeles, CA 90033, USA; albertopuccini91@gmail.com (A.P.); lenz@med.usc.edu (H.-J.L.); fbattagl@usc.edu (F.B.); 3 Caris Life Sciences, Phoenix, AZ 85040, USA; jxiu@carisls.com (J.X.); ybaca@carisls.com (Y.B.); wmkorn@carisls.com (W.M.K.); 4 Department of Internal Medicine, Oncologic Day Hospital, Bressanone Hospital (SABES-ASDAA), 39042 Bressanone-Brixen, Italy; 5 West Virginia University Cancer Institute, Morgantown, WV 26506, USA; Richard.goldberg@hsc.wvu.edu; 6 West Cancer Center, Germantown, TN 38138, USA; agrothey@westclinic.com; 7 Department of Oncology, Karmanos Cancer Institute, Wayne State University, Detroit, MI 48201, USA; shieldsa@karmanos.org; 8 Levine Cancer Institute, Charlotte, NC 28204, USA; Mohamed.salem@carolinashealthcare.org; 9 Ruesch Center for the Cure of Gastrointestinal Cancers, Lombardi Comprehensive Cancer Center, Georgetown University Medical Center, Washington, DC 20057, USA; marshalj@georgetown.edu

**Keywords:** *WRN*, colorectal cancer, MSI-H/dMMR, TMB, PD-L1, BRCAness, molecular profiling, immunotherapy

## Abstract

Werner syndrome gene (*WRN*) contributes to DNA repair. In cancer, *WRN* mutations (*WRN*-mut) lead to genomic instability. Thus, *WRN* is a promising target in cancers with microsatellite instability (MSI). We assessed this study to investigate the molecular profile of *WRN*-mut in colorectal cancer (CRC). Tumor samples were analyzed using next-generation sequencing (NGS) in-situ hybridization and immunohistochemistry. Tumor mutational burden (TMB) was calculated based on somatic nonsynonymous missense mutations. Determination of tumor mismatch repair (MMR) or microsatellite instability (MSI) status was conducted by fragment analysis. *WRN*-mut were detected in 80 of 6854 samples (1.2%). *WRN*-mut were more prevalent in right-sided compared to left-sided CRC (2.5% vs. 0.7%, *p* < 0.0001). TMB, PD-L1 and MSI-H/dMMR were significantly higher in *WRN*-mut than in *WRN* wild-type (WRN-wt). WRN-mut were associated with a higher TMB in the MSI-H/dMMR and in the MSS (microsatellite stable) subgroups. Several genetic differences between *WRN*-mut and *WRN*-wt CRC were observed, i.e., *TP53* (47% vs. 71%), *KRAS* (34% vs. 49%) and *APC* (56% vs. 73%). This is the largest molecular profiling study investigating the genetic landscape of *WRN*-mut CRCs so far. A high prevalence of MSI-H/dMMR, higher TMB and PD-L1 in *WRN*-mut tumors were observed. Our data might serve as an additional selection tool for trials testing immune checkpoint antibodies in *WRN*-mut CRC.

## 1. Introduction

Colorectal cancer (CRC) is the second most diagnosed cancer in women and the third in men. It is estimated that in 2018, 1.8 million patients were diagnosed with CRC and almost 861,000 deaths were attributed to CRC worldwide [1]. Considering this, there is an unmet clinical need for improvement of therapy. Despite advances in genomics, transcriptomics and proteomics, the translation of these findings into clinical routine treatment regimens in CRC has been rarely successful. Treatment with inhibitors of the immune checkpoint Programmed Death-1 (PD-1)/Programmed Death-Ligand 1 (PD-L1) led to substantial improvements in the prognosis of metastatic melanoma [2] and lung cancer patients [3]. In CRC, only a subset of patients, namely those with a damaged DNA mismatch repair gene and/or a microsatellite instability (MSI-H/dMMR), seem to benefit from a treatment with checkpoint inhibitors [4,5]. An MSI-H/dMMR state is observed in approximately 5–15% of all sporadic CRC cases [6,7,8]. MSI is usually the result of a hypermethylation of the MLH1 promotor or mutations in other MMR (mismatch repair) genes such as MSH2 [9].

In contrast to microsatellite stable (MSS) CRCs, MSI-H/dMMR CRCs have a distinct clinicopathologic phenotype, which has been described in the consensus molecular subtype (CMS) as the ‘CMS1/MSI immune’ type. This subtype can be distinguished by its hypermutated genotype and immune cell infiltration from the ‘CMS2/canonical’, ‘CMS3/metabolic’ and ‘CMS4/mesenchymal’ types [10].

In two recently published studies, ‘*Werner Syndrome RecQ Like Helicase*’ (*WRN*) was identified as a new potential target in CRC [11,12]. *WRN* encodes for a DNA helicase that is critical for maintaining genomic stability. A biallelic germline inactivation of *WRN* leads to ‘Werner Syndrome’, an autosomal-recessive inherited progeria characterized by premature aging but also by a predisposition for the development of malignancies [13,14]. In tumor cell lines, the co-occurrence of *WRN* inactivation and MSI leads to cell death and cell cycle arrest via the acquisition of double-strand breaks and chromosomal instability. In contrast, in MSS cell lines, *WRN* was dispensable for cell survival [11]. These observations laid the foundation for concepts of developing a specific *WRN* inhibitor to induce synthetic lethality in MSI CRC. In this study we aimed to define the frequency of *WRN* mutations in a large CRC patient cohort and describe their impact on the overall molecular profile of *WRN*-mutated CRC.

## 2. Results

### 2.1. Incidence of WRN Mutations in Colorectal Cancer

*WRN* mutations (*WRN*-mut) were observed in 80 of 6854 samples (1.2%, see Table 1). Around 57% (*n* = 3905) of the specimens tested were obtained from the primary site of the tumor, whereas 43% (*n* = 2949) were samples derived from metastasis. A higher prevalence of *WRN*-mut was detected in primary tumor samples (1.3% vs. 0.9%, *p* = 0.0034). No differences were observed regarding age (*WRN*-wt 60.3 years vs. *WRN*-mut 62.5 years) or sex (female 1.2% vs. male 1.1%). *WRN* mutations were found more frequently in right-sided than in left-sided cancers (5.4% vs. 0.7%, *p* < 0.0001).

### 2.2. Molecular Portrait of WRN-Mutated and Wild-Type Colorectal Cancer

**Figure 1:**
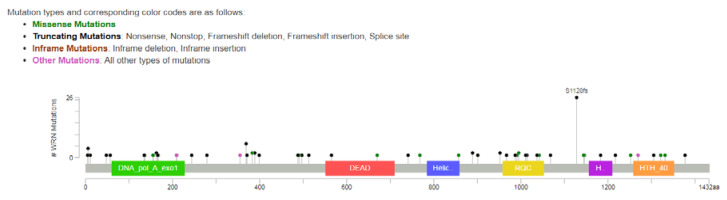
Location of the detected mutations in the *WRN* (Warner syndrome) gene. A black dot indicates a truncating mutation (nonsense, frameshift mutations and mutations at the splice sites); the blue dots indicate a truncating mutation, for which the exact effect could not be determined. No mutations could be detected in the helicase domain. Figure created with the ‘cbioportal mutation mapper’ (https://www.cbioportal.org/mutation_mapper).

The most frequently observed *WRN* gene alteration was the S1128fs frameshift mutation, contributing to 30.9% of the detected mutations, followed by the R369X nonsense mutation (7.1%) and the L6fs frameshift mutation (4.7%). No mutations were observed in the helicase domain (see Figure 1). Other mutations were detected at a much lower rate and a full list is provided in Table S1.

**Figure 2:**
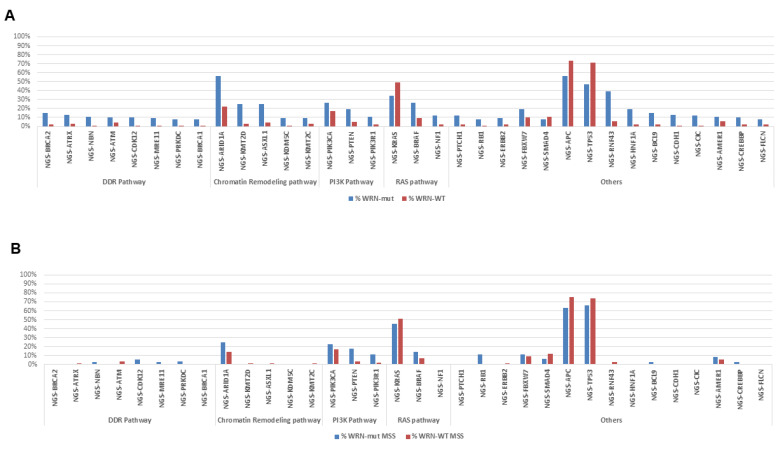
Molecular landscape of gene alterations in *WRN*-mut (WRN mutations) vs. *WRN*-wt (WRN wild type) statistically significant biomarkers in (**A**) all samples and (**B**) MSS only samples.

Several differences between *WRN*-mut and *WRN*-wt metastatic CRC (mCRC) samples were observed (Figure 2). *WRN*-mut tumors were characterized by a significantly different prevalence of co-mutations of the following genes: *ARID1A* (56% vs. 22%), *APC* (56% vs. 73%), and *TP53* (47% vs. 71%), *RNF43* (39% vs. 6%), *KRAS* (34% vs. 49%), and *BRAF* (26% vs. 9%) (all *p* < 0.01). Furthermore, a higher proportion of ‘BRCAness’ genes were detected in *WRN*-mut cases: *BRCA1* (8% vs. 1%), *BRCA2* (15% vs. 2%), and *ATM* (10% vs. 4%). Additionally, copy number alterations (CNA) of *CDX2* were only seen in *WRN*-wt tumors (6% vs. 0%, *p* = 0.027). The following CNAs were also more frequently detected in *WRN*-mut CRC: *CD274*, *CALR*, *CRTC1*, *ELL*, *JAK3*, *KEAP1*, *LYL1* and *MEF2B* (*p* < 0.01).

### 2.3. WRN Mutations Correlate with PDL1, TMB and MSI-H/dMMR

**Figure 3:**
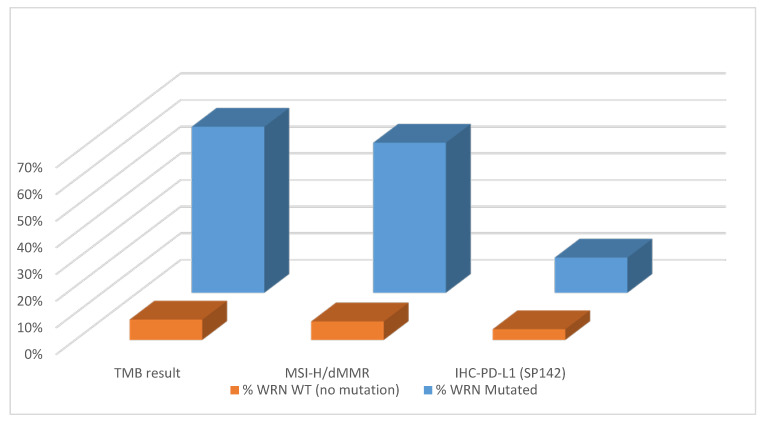
Tumor mutational burden (TMB), microsatellite instability-high/mismatch repair system deficient (MSI-H/dMMR) and Programmed Death-ligand 1 (PD-L1) in *WRN*-mut (orange) vs. *WRN*-wt (blue) cases.

In *WRN*-mut CRC, in contrast to *WRN*-wt, a higher co-occurrence of MSI-H/dMMR was observed (56% vs. 7%, *p* < 0.0001, Figure 3). *WRN*-mut CRCs were associated with a higher mean tumor mutational burden (TMB) (49 vs. 10.7 mutations/megabase [mut/MB], *p* < 0.0001) and a higher PD-L1 expression (13% vs. 4%, *p* < 0.0001) compared to *WRN*-wt.

**Figure 4:**
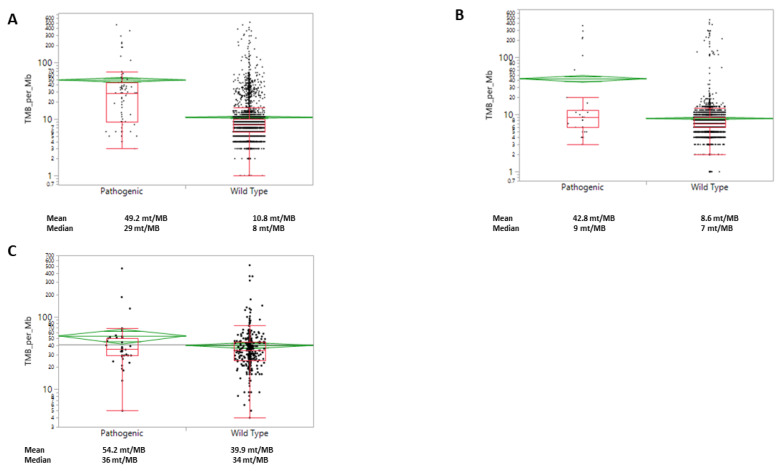
WRN mutations are significantly associated with an increased TMB in colorectal cancer. (**A**) All samples; (**B**) MSS (microsatellite stable) samples; (**C**) in MSI-H/dMMR samples.

Within the MSI-H/dMMR subgroup, *WRN*-mut was associated with a higher mean TMB (54 vs. 40 mut/MB, *p* = 0.03, Figure 4). Similar observations were made in the MSS subgroup, where a higher mean TMB was seen in *WRN*-mut when compared to *WRN*-wt cases (43 vs. 8.6 mut/MB, *p* < 0.0001). However, when looking at median levels, the differences observed with mean levels are no longer statistically significant.

## 3. Discussion

To the best of our knowledge, this analysis represents the largest study investigating somatic *WRN* mutations and co-occurring genomic alterations in CRC. Overall, in this unselected CRC cohort, *WRN*-mut were identified in 1.2% of analyzed samples. In a previous study using data from the Cancer Genome Atlas, 4% (20 out of 224 patients) showed a WRN mutation [15]. However, in this smaller population, a mixture of gene alterations (loss-of-function and missense mutations with uncharacterized functions) was included. Our study, on the other hand, was strictly restricted to loss-of-function events.

Tumors harboring a *WRN* mutation were characterized by a distinct molecular profile compared to *WRN*-wt samples.

We observed a higher incidence of *WRN*-mut tumors in patients with right-sided CRC and a lower frequency of mutations in *KRAS, APC* and *TP53*, as well as a higher rate of concomitant *BRAF* mutations, MSI-H/dMMR and mutations in other DDR-genes than in *WRN*-wt samples. Still, a significant proportion of samples was found to have mutations in the respective genes. It has been shown that right-sided CRC is more frequently induced by MSI-H/dMMR and *BRAF* mutations [16,17], whereas in left-sided CRC, the adenoma-carcinoma sequence is driven by the acquisition of *APC*, *KRAS* and *TP53* alterations [18,19]. Within this study, we could only demonstrate associations and thus, conclusions about possible causalities are merely hypothetical. However, the lower incidence of mutations in genes of the ‘CIN pathway’ in *WRN*-mut samples could indicate that mutations in *WRN* play a role in the evolution of these cancers. However, it is not known at which step in the carcinogenesis of CRC somatic *WRN* mutations occur and how they influence malignant transformation. To investigate such questions, further studies need to be conducted.

*BRCA1* and *BRCA2* mutations, as well as other ‘BRCAness’ describing gene alterations, were more frequently observed in *WRN*-mut samples than in wild-type cases. ‘BRCAness’ describes homologous recombination repair defects in the absence of a *germline BRCA1* or *BRCA2* mutation. This includes next to *somatic BRCA1/2* alterations, mutations in other genes such as the *ATM*, *ATR*, *CHEK1/2*, *ARID1A* and also *WRN* [20,21]. Since the approval of PARP inhibitors for *BRCA*-mutated breast and pancreatic cancer [22,23], researchers aim to translate these findings into other settings. For CRC, the PARP inhibitors olaparib and veliparib have been evaluated as monotherapy [24] or in combination therapy [25] with only modest efficacy. In some of these trials, patients were stratified according to MSI status. To the best of our knowledge, further stratification regarding ‘BRCAness’ status was not performed. We hypothesize that patients with *WRN*-mut CRC may also benefit from PARP inhibitor treatment [21].

In our cohort, MSI-H/dMMR status was observed in 56% of WRN-mut tumors, whereas it was only observed in 7% of *WRN*-wt tumors. Previous studies suggested a synthetic lethal effect of the inactivation of the helicase domain of *WRN* and concomitant MSI, leading to cell cycle arrest and to induction of apoptosis, mainly through impaired restoration of DNA double-strand breaks [11,12]. Our study was strictly restricted to loss-of-function events that were deemed pathogenic by board certified geneticists, which included nonsense, frameshift mutations and mutations that happen at the splice sites, causing loss of function of WRN proteins. However, we did not detect any mutation in the helicase domain, which was found to be essential for cell survival in vitro [11,12]. We hypothesize that cells with somatic truncating mutations in the helicase domain of the *WRN* gene and co-occurring MSI were not viable. A correlation between *WRN* promotor methylation and the CIMP phenotype has been described earlier, linking deficiency in *WRN* gene function and MSI [26].

As we did not perform RNomics, proteomics or protein function analysis, we cannot conclude on expression levels, state of heterozygosity or any function left of the truncated WRN proteins.

MSI-H/dMMR status has been identified as an independent predictor of response to PD-1 blockade by pembrolizumab [27], which was therefore approved as a site-agnostic drug by the FDA. Besides the MSI-H/dMMR status, TMB is currently evaluated as a further predictive biomarker for immune checkpoint inhibition in various cancers [28]. In a retrospective study of MSI-H/dMMR metastatic CRC featuring patients who underwent treatment with a checkpoint inhibitor, a TMB score of more than 41 mut/MB was determined as a predictive cut-off [29]. In our cohort, the median TMB in *WRN*-mut tumors was significantly higher (49 mut/MB) than in *WRN*-wt tumors (10.7 mut/MB). This may be explained by the role of the *WRN*-helicase as a critical player in the DNA repair system [30]. Taking the high rate of MSI-H/dMMR and the high TMB score into consideration, checkpoint inhibition might be an option in *WRN*-mut CRC. Additionally, *WRN*-mut tumors had a higher PD-L1 expression than *WRN*-wt tumors (13% vs. 4%, *p* < 0.0001), which is still under discussion as a biomarker of response [31]. In the setting of CRC, PD-L1 expression levels do not seem to play a major role in predicting response upon checkpoint therapy, as was observed in the Checkmate 142 trial [32].

Interestingly, we also observed more *RNF43* mutations in *WRN*-mut samples (39%) than in *WRN*-wt tumors (6%, *p* < 0.01). *RNF43* mutations, next to R-spondin fusion proteins, lead to an activation of the WNT-signaling pathway [33,34] that represents one of the crucial pathways in CRC. Somatic *RNF43* mutations have been described to be present in about 3% of CRC and were strongly associated with an MSI-H/dMMR phenotype [33]. To the best of our knowledge, so far no one has described an association between *WRN* and *RNF43*. We hypothesize that there is no direct interaction between these proteins and that the higher incidence is induced secondarily by the MSI-H/dMMR status.

As described recently, the helicase domain of *WRN* is considered as a therapeutic target for synthetic lethality, as the exonuclease domain is dispensable for cell survival in MSI-H tumor cells. Thus, inhibiting *WRN* may be an attractive target for MSI-H/dMMR tumors. Inhibitors, such as NSC19630 [35], NSC617145 [36], ML216 [37], NCGC00029283, NCGC00063279, and NCGC00357377 [38] have been identified in drug-screening studies [39]; however, none of them are in a clinical trial yet.

Chan and colleagues [11] also investigated the dependency of *WRN* depletion on *TP53* status. They found that *TP53*-wt/MSI-H tumor cells were more sensitive to *WRN* loss than *TP53*-mut/MSI-H cells; they also found an increased expression of the CDK inhibitor *p21* subsequent to a *WRN* depletion, which is a response to *TP53* activation. For the success of *WRN* inhibitors, it could be essential to assess, besides MSI status, *TP53* mutations as a biomarker of response, as a mutated *TP53* could impair efficacy of *WRN* inhibition.

Our study has important limitations, such as retrospective data extraction from a large database including only very limited basic clinical data. However, we think that the data set is sufficient to initiate future in vitro and in vivo experiments as well as to use the *WRN* mutation status for biomarker-driven clinical trials testing PARP and checkpoint inhibitors. Moreover, it would be important to define the frequency of *WRN* mutations in other tumor entities.

## 4. Material and Methods

Specimens of 6854 colorectal cancer patients were sent to Caris Life Sciences between March 2015 and March 2019. Patients’ consent included specimen submission and molecular profiling but did not include access to medical records. Thus, only basic demographic information was available. This study was conducted in accordance with guidelines of the Declaration of Helsinki, Belmont report and U.S. Common rule. In keeping with 45 CFR 46.101(b)(4), this study was performed utilizing retrospective, deidentified clinical data. Thus, this study is considered IRB exempt and no patient consent was necessary from the subjects. Patients were stratified into *WRN*-mutated and *WRN*-wildtype cases. The *WRN* mutations included only pathogenic or presumably pathogenic mutations. Benign, presumed benign *WRN* mutation or WRN variants of unknown significance were categorized as *WRN* wild-type. Statistical comparison was performed with the Chi-square test.

Immunohistochemistry (IHC) was performed on 6854 tumor samples on formalin-fixed paraffin-embedded (FFPE) sections on glass slides. Four µm sections mounted on slides were stained using an automated system (Benchmark, Ventana Medical Systems, Tucson, AZ; Autostainer, DAKO, Carpinteria, CA) according to manufacturer’s instructions and were optimized and validated per CLIA/CAO and ISO requirements. All proteins of interest were evaluated on tumor cells. An intensity score (0 = no staining; 1+ = weak staining; 2+ = moderate staining; 3+ = strong staining) and a proportion score to determine the percentage of cells staining positive (0–100%) were used. The primary antibody used against PD-L1 was SP142 (Spring Biosciences). The staining was deemed positive if its intensity on the membrane of the tumor cells was ≥2+ and the percentage of positively stained cells was ≥5%. Immunohistochemical results were independently evaluated by a board-certified pathologist.

Next-generation sequencing (NGS) was performed on 6854 tumor samples on genomic DNA isolated from FFPE tumor samples using the NextSeq platform (Illumina, Inc., San Diego, CA, USA). A custom-designed SureSelect XT assay was used to enrich 592 whole-gene targets (Agilent Technologies, Santa Clara, CA, USA). The full list of genes sequenced are illustrated in the (Supplemental Materials Table S2). All variants were detected with >99% confidence based on allele frequency and amplicon coverage with an average sequencing depth of coverage of >500 and with an analytic sensitivity of 5%. Variants detected were mapped to reference genome (hg19) and well-established bioinformatics tools such as BWA, SamTools, GATK and snpFF were incorporated to perform variant calling functions; germline variants were filtered with various germline databases including 1000 genome and dbSNP. Genetic variants identified were interpreted by board-certified molecular geneticists and categorized as ‘pathogenic,’ ‘presumed pathogenic,’ ‘variant of unknown significance,’ ‘presumed benign’ or ‘benign,’ according to American College of Medical Genetics and Genomics (ACMG) standards. Pathogenic mutations included nonsense, frameshift mutations and mutations that happen at the splice sites, causing loss of function of WRN proteins. When assessing mutation frequencies of individual genes, ’pathogenic’ and ‘presumed pathogenic’ were defined as mutations whereas ‘benign’ or ‘presumed benign’ variants and ‘variants of unknown significance’ were excluded.

Tumor mutational burden was measured (592 genes and 1.4 megabases (MB) sequenced per tumor) by counting all non-synonymous missense mutations found per tumor that had not been previously described as germline alterations. A combination of multiple test platforms was used to determine the MSI or MMR status of the tumors profiled, including fragment analysis (FA, Promega, Madison, WI, USA), IHC (MLH1, M1 antibody; MSH2, G2191129 antibody; MSH6, 44 anti-body; and PMS2, EPR3947 antibody (Ventana Medical Systems, Inc., Tucson, AZ, USA)) and NGS (for tumors tested with the NextSeq platform, 7000 target microsatellite loci were examined and compared to the reference genome hg19 from the University of California).

Patient and molecular characteristics of WRN-mutated and -wild-type tumors were compared using standard statistical tests. In the comparison, the age was analyzed by Student *t*-test and the TMB distribution was tested by Nonparametric Kruskal-Wallis testing. The other categorical data were analyzed by Fisher’s exact or Chi-Square tests. Cases with missing information in any of its data were not included in the analysis. All tests were two-sided at a significance level of 0.05. As this is an exploratory study, no correction for multiple comparison was performed.

## 5. Conclusions

This is the largest profiling study investigating the molecular landscape of *WRN*-mut CRC. We show an association between MSI-H/dMMR status and *WRN*-mutation as well as a link to higher TMB and PD-L1 expression. However, the clinical correlate of an incidental response of non MSI-H/dMMR tumors to checkpoint antibodies suggests that other rare genetic subtypes may predispose response to immune checkpoint therapy. *WRN*-mut CRC may be such a predisposing phenotype, as it is also linked to high TMB and PD-L1 expression. Finally, *WRN*-mut CRC is characterized by a distinct genetic profile of co-mutations and BRCA-ness. Our data might set the stage for testing PARP and checkpoint inhibition in *WRN*-mut CRC.

## Figures and Tables

**Table 1:** Demographical characteristics.

		WRN—No Mutation	WRN—Mutation	Mutation %	*p* Value
**Specimen Type**	Metastasis	2921	28	0.9%	0.0034
	Primary/local	3853	52	1.3%	
**Total**		6774	80	1.2%	–
**Age**	Median age	60.3	62.5	–	NS (not significant)
**Gender**	Female	3062	38	1.2%	NS
	Male	3712	42	1.1%	
**Sidedness**	Left	3371	24	0.7%	*p* < 0.0001
	Right	1743	44	2.5%	
	NOS (not otherwise specified)	1660	12	0.7%	

## Supplementary Materials

The following are available online at https://www.mdpi.com/2072-6694/12/5/1319/s1, Table S1: Mutations in the *WRN* gene, Table S2: List of the 592 genes analyzed.
