# Supplementary material for: WRN-Mutated Colorectal Cancer Is Characterized by a Distinct Genetic Phenotype
Source: Cancers (Basel). 2020 May 22;12(5):1319. doi: 10.3390/cancers12051319 (PMC7281075; doi:10.3390/cancers12051319)
Supplement: Supplementary file 1 [file cancers-12-01319-s001.pdf]

cancers

MDPI

Article

WRN-Mutated Colorectal Cancer Is Characterized by a Distinct Genetic Phenotype

Kai Zimmer, Alberto Puccini, Joanne Xiu, Yasmine Baca, Gilbert Spizzo, Heinz-Josef Lenz, Francesca Battaglin, Richard M. Goldberg, Axel Grothey, Anthony F. Shields, Mohamed E. Salem, John L. Marshall, W. Michael Korn, Dominik Wolf, Florian Kocher and Andreas Seeber

Supplementary Material

Table S1. Mutations in the *WRN* gene.

Protein Change	N (%)	Effect of Mutation
S1128fs	26 (30.9)	Frameshift
R369X	6 (7.1)	Nonsense
Frameshift*	4 (4.7)	
L6fs	4 (4.7)	Frameshift
D163fs	2 (2.38)	Frameshift
R389fs	2 (2.38)	Frameshift
R889X	2 (2.38)	NA
S952X	2 (2.38)	Nonsense
A136fs	1 (1.19)	Frameshift
c.1269+2T>C	1 (1.19)	NA
c.1898+2T>G	1 (1.19)	NA
c.209+2T>A	1 (1.19)	Splicing
c.210-1G>A	1 (1.19)	Splicing
c.3383+2T>C	1 (1.19)	NA
c.355+1G>T	1 (1.19)	Splicing
c.3687+2T>G	1 (1.19)	Splicing
c.3983-1G>T	1 (1.19)	Splicing
E1068X	1 (1.19)	Nonsense
E1217X	1 (1.19)	Nonsense
E244X	1 (1.19)	Nonsense
E371X	1 (1.19)	NA
E399X	1 (1.19)	Nonsense
E488X	1 (1.19)	Nonsense
E48X	1 (1.19)	Nonsense
E513X	1 (1.19)	Frameshift
F1037fs	1 (1.19)	Frameshift
G1377fs	1 (1.19)	Frameshift
I1183fs	1 (1.19)	Frameshift
K135fs	1 (1.19)	Nonsense
K167X	1 (1.19)	Frameshift
K5fs	1 (1.19)	Frameshift
K901fs	1 (1.19)	Frameshift
L967fs	1 (1.19)	Frameshift
M497fs	1 (1.19)	Frameshift

N166fs	1 (1.19)	Frameshift
Q11fs	1 (1.19)	Frameshift
R1305X	1 (1.19)	Nonsense
R279fs	1 (1.19)	Frameshift
R565X	1 (1.19)	Nonsene
R741X	1 (1.19)	Nonsense
R987X	1 (1.19)	NA
T1011fs	1 (1.19)	Frameshift
W1014X	1 (1.19)	Nonsense
Y57X	1 (1.19)	Nonsense
Grand Total	84	

*4 Frameshift mutations could not be described more precisely.

Table S2. List of the 592 genes analyzed.

ABI1
ABL1
ABL2
ACKR3
ACSL3
ACSL6
ADGRA2
AFDN
AFF1
AFF3
AFF4
AKAP9
AKT1
AKT2
AKT3
ALDH2
ALK
AMER1
APC
AR
ARAF
ARFRP1
ARHGAP26
ARHGEF12
ARID1A
ARID2
ARNT
ASPSCR1
ASXL1
ATF1
ATIC
ATM
ATP1A1
ATP2B3
ATR
ATRX
AURKA

AURKB
AXIN1
AXL
BAP1
BARD1
BCL10
BCL11A
BCL11B
BCL2
BCL2L11
BCL2L2
BCL3
BCL6
BCL7A
BCL9
BCOR
BCORL1
BCR
BIRC3
BLM
BMPR1A
BRAF
BRCA1
BRCA2
BRD3
BRD4
BRIP1
BTG1
BTK
BUB1B
C15orf65
CACNA1D
CALR
CAMTA1
CANT1
CARD11
CARS
CASP8
CBFA2T3
CBFB
CBL
CBLB
CBLC
CCDC6
CCNB1IP1
CCND1
CCND2
CCND3
CCNE1
CD274
CD74
CD79A

CD79B
CDC73
CDH1
CDH11
CDK12
CDK4
CDK6
CDK8
CDKN1B
CDKN2A
CDKN2B
CDKN2C
CDX2
CEBPA
CHCHD7
CHEK1
CHEK2
CHIC2
CHN1
CIC
CIITA
CLP1
CLTC
CLTCL1
CNBP
CNOT3
CNTRL
COL1A1
COPB1
COX6C
CREB1
CREB3L1
CREB3L2
CREBBP
CRKL
CRLF2
CRTC1
CRTC3
CSF1R
CSF3R
CTCF
CTLA4
CTNNA1
CTNNB1
CYLD
CYP2D6
DAXX
DDB2
DDIT3
DDR2
DDX10
DDX5

DDX6
DEK
DICER1
DNM2
DNMT3A
DOT1L
EBF1
ECT2L
EGFR
EIF4A2
ELF4
ELK4
ELL
ELN
EML4
EMSY
EP300
EPHA3
EPHA5
EPHB1
EPS15
ERBB2
ERBB3
ERBB4
ERC1
ERCC1
ERCC2
ERCC3
ERCC4
ERCC5
ERG
ESR1
ETV1
ETV4
ETV5
ETV6
EWSR1
EXT1
EXT2
EZH2
EZR
FAM46C
FANCA
FANCC
FANCD2
FANCE
FANCF
FANCG
FANCL
FAS
FBXO11
FBXW7

FCRL4
FEV
FGF10
FGF14
FGF19
FGF23
FGF3
FGF4
FGF6
FGFR1
FGFR1OP
FGFR2
FGFR3
FGFR4
FH
FHIT
FIP1L1
FLCN
FLI1
FLT1
FLT3
FLT4
FNBP1
FOXA1
FOXL2
FOXO1
FOXO3
FOXO4
FOXP1
FSTL3
FUBP1
FUS
GAS7
GATA1
GATA2
GATA3
GID4
GMPS
GNA11
GNA13
GNAQ
GNAS
GOLGA5
GOPC
GPC3
GPHN
GRIN2A
GSK3B
H3F3A
H3F3B
HERPUD1
HEY1

HGF
HIP1
HIST1H3B
HIST1H4I
HLF
HMGA1
HMGA2
HMGN2P46
HNF1A
HNRNPA2B1
HOOK3
HOXA11
HOXA13
HOXA9
HOXC11
HOXC13
HOXD11
HOXD13
HRAS
HSP90AA1
HSP90AB1
IDH1
IDH2
IGF1R
IKBKE
IKZF1
IL2
IL21R
IL6ST
IL7R
INHBA
IRF4
IRS2
ITK
JAK1
JAK2
JAK3
JAZF1
JUN
KAT6A
KAT6B
KCNJ5
KDM5A
KDM5C
KDM6A
KDR
KDSR
KEAP1
KIAA1549
KIF5B
KIT
KLF4

KLHL6
KLK2
KMT2A
KMT2C
KMT2D
KNL1
KRAS
KTN1
LASP1
LCK
LCP1
LGR5
LHFPL6
LIFR
LMO1
LMO2
LPP
LRIG3
LRP1B
LYL1
MAF
MAFB
MALT1
MAML2
MAP2K1
MAP2K2
MAP2K4
MAP3K1
MAX
MCL1
MDM2
MDM4
MDS2
MECOM
MED12
MEF2B
MEN1
MET
MITF
MKL1
MLF1
MLH1
MLLT1
MLLT10
MLLT11
MLLT3
MLLT6
MN1
MNX1
MPL
MRE11
MSH2

MSH6
MSI2
MSN
MTCP1
MTOR
MUC1
MUTYH
MYB
MYC
MYCL
MYCN
MYD88
MYH11
MYH9
NACA
NBN
NCKIPSD
NCOA1
NCOA2
NCOA4
NDRG1
NF1
NF2
NFE2L2
NFIB
NFKB2
NFKBIA
NIN
NKX2-1
NONO
NOTCH1
NOTCH2
NPM1
NR4A3
NRAS
NSD1
NSD2
NSD3
NT5C2
NTRK1
NTRK2
NTRK3
NUMA1
NUP214
NUP93
NUP98
NUTM1
NUTM2B
OLIG2
OMD
P2RY8
PAFAH1B2

PAK3
PALB2
PATZ1
PAX3
PAX5
PAX7
PAX8
PBRM1
PBX1
PCM1
PCSK7
PDCD1
PDCD1LG2
PDE4DIP
PDGFB
PDGFRA
PDGFRB
PDK1
PER1
PHF6
PHOX2B
PICALM
PIK3CA
PIK3CG
PIK3R1
PIK3R2
PIM1
PLAG1
PML
PMS1
PMS2
POLE
POT1
POU2AF1
POU5F1
PPARG
PPP2R1A
PRCC
PRDM1
PRDM16
PRF1
PRKAR1A
PRKDC
PRRX1
PSIP1
PTCH1
PTEN
PTPN11
PTPRC
RABEP1
RAC1
RAD21

RAD50
RAD51
RAD51B
RAF1
RALGDS
RANBP17
RAP1GDS1
RARA
RB1
RBM15
RECQL4
REL
RET
RHOH
RICTOR
RMI2
RNF213
RNF43
ROS1
RPL10
RPL22
RPL5
RPN1
RPTOR
RUNX1
RUNx1T1
SBDS
SDC4
SDHAF2
SDHB
SDHC
SDHD
SEPT5
SEPT6
SEPT9
SET
SETBP1
SETD2
SF3B1
SFPQ
SH2B3
SH3GL1
SLC34A2
SLC45A3
SMAD2
SMAD4
SMARCA4
SMARCB1
SMARCE1
SMO
SNX29
SOCS1

SOX10
SOX2
SPECC1
SPEN
SPOP
SRC
SRGAP3
SRSF2
SRSF3
SS18
SS18L1
SSX1
STAG2
STAT3
STAT4
STAT5B
STIL
STK11
SUFU
SUZ12
SYK
TAF15
TAL1
TAL2
TBL1XR1
TCEA1
TCF12
TCF3
TCF7L2
TCL1A
TERT
TET1
TET2
TFE3
TFEB
TFG
TFPT
TFRC
TGFBR2
THRAP3
TLX1
TLX3
TMPRSS2
TNFAIP3
TNFRSF14
TNFRSF17
TOP1
TP53
TPM3
TPM4
TPR
TRAF7

TRIM26
TRIM27
TRIM33
TRIP11
TRRAP
TSC1
TSC2
TSHR
TTL
U2AF1
UBR5
USP6
VEGFA
VEGFB
VHL
VTI1A
WAS
WDCP
WIF1
WISP3
WRN
WT1
WWTR1
XPA
XPC
XPO1
YWHAE
ZBTB16
ZMYM2
ZNF217
ZNF331
ZNF384
ZNF521
ZNF703
ZRSR2
